# IL-6: A Potential Role in Cardiac Metabolic Homeostasis

**DOI:** 10.3390/ijms19092474

**Published:** 2018-08-21

**Authors:** Yitao Xu, Yubin Zhang, Junmei Ye

**Affiliations:** 1 Division of Cancer, Department of Surgery and Cancer, Imperial College London, London W120NN, UK; yito-xu@outlook.com; 2 State Key laboratory of Natural Medicines, Department of Biochemistry, School of Life Science and Technology, China Pharmaceutical University, Nanjing 210006, China

**Keywords:** interleukin-6 (IL-6), lipid metabolism, cardiac lipotoxicity, mitochondrial dysfunction

## Abstract

Interleukin-6 (IL-6) is implicated in multiple biological functions including immunity, neural development, and haematopoiesis. Recently, mounting evidence indicates that IL-6 plays a key role in metabolism, especially lipid metabolic homeostasis. A working heart requires a high and constant energy input which is largely generated by fatty acid (FA) β-oxidation. Under pathological conditions, the precise balance between cardiac FA uptake and metabolism is perturbed so that excessive FA is accumulated, thereby predisposing to myocardial dysfunction (cardiac lipotoxicity). In this review, we summarize the current evidence that suggests the involvement of IL-6 in lipid metabolism. Cardiac metabolic features and consequences of myocardial lipotoxicity are also briefly analyzed. Finally, the roles of IL-6 in cardiac FA uptake (i.e., serum lipid profile and myocardial FA transporters) and FA metabolism (namely, β-oxidation, mitochondrial function, biogenesis, and FA *de novo* synthesis) are discussed. Overall, understanding how IL-6 transmits signals to affect lipid metabolism in the heart might allow for development of better clinical therapies for obesity-associated cardiac lipotoxicity.

## 1. Introduction

Due to the functional pleiotropy of cytokines, interleukin-6 (IL-6) is involved in a variety of biological processes. IL-6 was originally considered to play a role in acute phase of inflammation since its expression is induced in response to viral infection [1,2,3], lipopolysaccharide [4], and other cytokines [5,6]. Later, it was reported that IL-6 is necessary for switching from neutrophils to monocytes [7,8,9,10], antibody production by B-cells [11], as well as recruitment and differentiation of T-cells [12,13]. Moreover, other biological functions unrelated to immune system including neural development [14], liver regeneration [15,16], and bone homeostasis [17] have been demonstrated.

Recently, emerging evidence suggests a key role of IL-6 in metabolism. Wallenius et al. reported that IL-6 deficiency in mice results in the development of mature-onset obesity which is partially reserved by intracerebroventricular IL-6 injection [18]. Furthermore, mice overexpressing IL-6 showed reduced body weight which correlates well with decreased fat mass [19]. Of note, infusion of IL-6 leads to enhanced lipolysis in skeletal muscle and increased systemic fatty acid (FA) oxidation in humans [20]. Taken together, these data indicate a link between IL-6 and metabolism—in particular, lipid metabolism.

Normally, cardiac energy demands are largely met by FA β-oxidation [21]. However, intramyocardial accumulation of lipid due to an imbalance between lipid uptake and oxidation leads to cardiac dysfunction, which is termed lipotoxicity [22]. Consequently, FA deposits and its toxic metabolites elicit ER (Endoplasmic Reticulum) stress, mitochondria dysfunction, and apoptosis, thereby impairing cardiac contractile function and predisposing to various cardiomyopathies (e.g., heart failure and arrhythmia) [23]. However, little is understood about the role of IL-6 in cardiac metabolism and lipotoxicity. The article reviews the importance of IL-6 in cardiac metabolic homeostasis and possible underlying mechanisms, hoping to provide new insights into therapeutic potential of IL-6 in targeting cardiac diseases caused by metabolic disorder.

## 2. The Role of IL-6 in Lipid Metabolism

Several lines of evidence suggest a role of IL-6 in metabolic control in humans (Table 1). Acute elevation of IL-6 plasma concentration enhances lipolysis in skeletal muscle and stimulates systemic FA oxidation [20]. Lyngsø et al. reported that IL-6 infusion leads to an increase of net glycerol release from subcutaneous adipose tissue and stimulates FA as well as glycerol uptake in splanchnic tissue in healthy humans, indicating that IL-6 promotes lipolysis in adipose tissue and affects metabolism in splanchnic tissue [24]. Moreover, Carey et al. [25] and Pederson et al. [26] showed that acute IL-6 treatment leads to increased lipid metabolism in vivo. Another study by Hall et al. demonstrated that both high-dose and low-dose infusion of recombinant human IL-6 (rhIL-6) stimulate lipolysis and result in elevation of global FA oxidation [27]. Of note, no alteration of plasma epinephrine, insulin, or glucagon was observed in the low-dose rhIL-6 infusion group, supporting the conclusion that the increased lipolysis and fat oxidation are primary effects of rhIL-6 administration. Furthermore, IL-6 neutralizing antibody Actemra (tocilizumab) induces body weight gain, hypertriglyceridemia, and hypercholesterinemia in humans [28]. Interestingly, carriers of *IL-6* gene polymorphism G174C exhibit a trend towards increased plasma IL-6 levels which are correlated with elevated plasma triglycerides (TG), very-low-density lipoprotein (VLDL)-triglycerides, and free fatty acid (FFA) [29]. The lack of significance of IL-6 levels in this study might result from technical difficulties in detecting both free IL-6 and that bound to carrier proteins.

The direct effects of IL-6 on metabolism are better elucidated by in vitro studies. Abdominal adipose tissue from overweight and obese humans cultured with IL-6 alone exhibits enhanced lipolysis [30]. Moreover, IL-6 treatment stimulates lipolysis in differentiated 3T3-L1 cells with adipocyte-like phenotype from mouse embryonic 3T3 fibroblasts [26]. In addition, acute IL-6 treatment stimulates FA oxidation in the cell line of rat L6 myotubes [26]. Another study using primary human skeletal muscle cells demonstrated that IL-6 treatment induces FA uptake and oxidation [31]. Taken together, the effects on metabolic homeostasis observed in these studies are more likely to be exerted by IL-6 per se.

Studies involving rodents also implicate a role of IL-6 in metabolic regulation. IL-6-deficient mice exhibit mature-onset obesity, with disrupted carbohydrate and lipid metabolism, which are partially reserved by intracerebroventricular (ICV) injection of rat IL-6 [18]. These authors went further and showed that chronic ICV injection of rat recombinant IL-6 reduces relative weight of mesenteric and retroperitoneal fat pads, thereby suggesting the anti-obesity role of IL-6 [32]. Mice over-expressing human IL-6 have less visceral fat when fed a normal diet and are free from high-fat-diet (HFD)-induced obesity [33]. Direct delivery of murine IL-6 via adenoviral vector into rat hypothalamus results in suppressed weight gain and reduction of visceral adiposity [34]. Furthermore, overexpression of mouse IL-6 by gene transfer in obese mice induces loss of body weight which correlates with reduction of fat mass and size of fat pads [19]. Interestingly, double-transgenic mice expressing both human IL-6 and human soluble IL-6 receptors (sIL-6R) exhibit more pronounced reduction of body weight and decrease of global fat in comparison with single-transgenic mice expressing IL-6 or IL-6 receptors [35]. Given the above results, IL-6 is suggested to play an anti-obesity role in rodent metabolic homeostasis (Table 2).

However, evidence against the anti-obesity role of IL-6 during HFD feeding has been reported. Our group showed a significantly more pronounced increase in body weight in wild-type (WT) mice in contrast to IL-6^−/−^ mice [36]. However, Sadagurski et al. created transgenic mice with sustained expression of human IL-6 and found that human IL-6 prevents body weight gain during HFD feeding [33]. Furthermore, Gregorio et al. failed to detect late-onset obesity or disturbed lipid metabolism in IL-6^−/−^ mice reported by Wallenius et al. [37]. Although it remains unclear, it is suggested that mice age, genetic background, strain specificity, potentially different environmental or dietary factors are likely to contribute to the discrepancy [36]. In addition, loss of body weight as well as decreased adiposity are usually late-stage symptoms caused by type 2 diabetes. In our study, impaired glucose tolerance and increased serum insulin level observed in IL-6^−/−^ mice were more severe than those in WT after HFD. Therefore, the anti-obesity effect of IL-6 deficiency during HFD could be a secondary symptom during HFD-induced obesity. Taken together, current evidence suggests that IL-6 is involved in metabolic regulation, especially lipid metabolism.

## 3. Cardiac Lipid Metabolism and Consequences of Lipotoxicity

**Figure 1:**
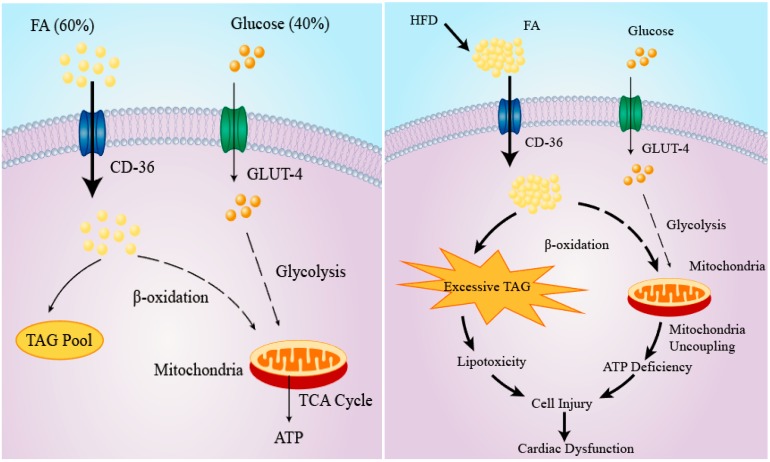
Schematic diagram of metabolism in cardiac myocyte. (**A**) Metabolism in normal cardiomyocytes. Under well-oxygenated conditions, cardiac energy requirement is largely met by fatty acid oxidation (~60%). Only a minor proportion (around 40%) is generated by glucose metabolism; and (**B**) Consequences of cardiac lipotoxicity. The precise balance between fatty acid uptake and oxidation is upset by metabolic disorders (i.e., HFD). Under these circumstances, excessive fatty acid is accumulated in cardiomyocytes and leads to cellular injury (e.g., mitochondria dysfunction, ER stress) and lipotoxicity, thereby predisposing to cardiac diseases (e.g., heart failure and arrhythmia). Dashed arrow: multistep processes; Solid arrow: direct effects. FA, fatty acid; TAG, triacylglyceride; GLUT-4, Glucose transporter type 4; TCA cycle, tricarboxylic acid cycle; HFD, high-fat diet; CD-36, cluster of differentiation 36 (also known as fatty acid translocase).

Under normal oxygenated conditions, a continuously contracting heart requires a high energy input which is primarily supplied by FA β-oxidation. Indeed, as much as 50–70% ATP generated in a heart is derived from FA β-oxidation [38,39]. Several key enzymes of glycolysis including hexokinases, phosphofructokinase (PFK), pyruvate dehydrogenase, as well as glucose transport are inhibited in well-perfused cardiac muscle, although the glycolytic pathway is stimulated in hypoxic and anoxic hearts [21] (Figure 1A).

The balance between cardiac FA uptake and β-oxidation is precisely maintained to ensure that no excess FA is accumulated in myocardium. However, metabolic disorders including obesity and diabetes tilt the balance towards enhanced uptake and/or reduced FA utilization [40,41]. Although excess FA can be kept in the heart as triglycerides (TG), the storage capacity is limited. Excessive FA accumulation is associated with dysfunction or even death of cardiomyocytes, which is termed as lipotoxicity [42]. Mitochondria dysfunction develops in hearts of ob/ob and db/db mice models. In these models, mitochondria are characterized by reduced ATP production despite enhanced FA β-oxidation, thereby suggesting mitochondrial uncoupling [43,44]. The impaired mitochondria coupling might result from stimulation of mitochondrial uncoupling protein by reactive oxygen species, the production of which is a marker of mitochondrial dysfunction [44]. Impaired cardiac insulin signaling due to toxic lipid metabolites (e.g., diacylglycerol (DAG) and ceramide) is another consequence of cardiac lipotoxicity [23]. Of note, cardiac insulin resistance in turn exacerbates lipotoxicity by disturbing cardiac glucose metabolism and augmenting FA uptake and accumulation [45]. Moreover, cardiac lipid deposition leads to apoptosis of cardiomyocytes and consequent systolic dysfunction in Zuker diabetic fatty rats [46]. Cardiac fibrosis develops following myocardial lipid accumulation, which increases the risk of cardiac arrhythmia and failure [47]. Furthermore, cardiac hypertrophy and increased mortality are linked to cardiac deposition of lipid in a transgenic mouse model overexpressing lipoprotein lipase (LPL) when fed a HFD [48]. Taken together, ectopic lipid accumulation in the heart interferes with myocardial function and thus predisposes to cardiovascular disease (Figure 1B).

## 4. The Role of IL-6 in Cardiac Lipotoxicity

Due to the central role of lipid in cardiac metabolism and the implication of IL-6 in lipid metabolic regulation, it is important to discuss the potential effects of IL-6 on cardiac lipotoxicity and underlying mechanisms. In this section, impacts of IL-6 on cardiac lipid supply, uptake, utilization, and *de novo* synthesis are elucidated.

### 4.1. IL-6, Dyslipidaemia, and FA Transporters

The potential link between IL-6 and dyslipidemia has been demonstrated by several studies. Since serum FA concentration is one of the major determinants of cardiac FA uptake rate [49], dyslipidemia secondary to IL-6 abnormality is likely to facilitate excessive FA import which may result in myocardial lipid accumulation and lipotoxicity. Our group noticed that IL-6 deficiency leads to increased levels of circulating TG and total cholesterol in female mice when fed an HFD [36]. Moreover, long-term administration of humanized anti-IL-6 antibody Actemra (tocilizumab) results in hypertriglyceridemia in human subjects and the severity appears to increase in a time-dependent manner [28]. Furthermore, IL-6 deficiency impairs hepatic insulin signaling pathway in chow-fed mice and exacerbates hepatic insulin resistance in mice fed HFD [50]. Since hepatic VLDL production is enhanced in an insulin-resistant state, it is suggested that IL-6 deficiency induces increased circulating TG concentration [51]. Elevated serum levels of TG and VLDL cholesterol were observed in IL-6^−/−^ female mice in comparison with WT female mice. However, no differences in plasma lipid profile were reported in IL-6^−/−^ male mice [18]. As stated above, these data could not be reproduced by other groups and the underlying reasons remain unclear [37]. Moreover, the correlation between IL-6 deficiency and dyslipidemia is challenged by one group reporting that IL-6 treatment is the causative factor of dyslipidemia. These authors showed that acute infusion of recombinant human IL-6 results in elevated plasma FA concentration in healthy human subjects without affecting circulating TAG levels [27]. The apparent discrepancy might result from experiment design in that long-term IL-6 deficiency achieved by IL-6 knockout and short-term IL-6 infusion may elicit different cellular responses, and thus may affect plasma lipid profiles differently. Taken together, current evidence indicates that IL-6 abnormality may lead to dyslipidemia which predisposes to cardiac lipotoxicity, although it remains unknown regarding which form (i.e., IL-6 excess or deficiency) is responsible and further research is required. 

Moreover, IL-6 deficiency upregulates expression of cardiac FA transporters. In comparison to passive diffusion, the majority of FA taken up by cardiomyocytes is mediated by protein carriers including fatty acid translocase (FAT/CD36), plasma membrane isoform of fatty acid binding protein (FABPpm), and fatty acid transport protein (FATP) 1/6, among which FAT/CD36 contributes to the translocation of 50–60% FFA [52,53]. Enhanced cardiac expression of FAT/CD36 was observed in IL-6^−/−^ mice with associated accumulation of biologically active lipids including FFA, DAG, and ceramide [54]. Our group also noticed that cardiac FAT/CD36 mRNA levels increase during HFD-induced obesity in both WT and IL-6^−/−^ mice [36]. However, the elevation is significantly more pronounced in IL-6^−/−^ mice, suggesting that IL-6 deficiency enhances FAT/CD36 expression. Considering the key role of FA transporters in cardiac lipid uptake, the regulatory effects of IL-6 on FAT/CD36 expression may have a more essential role in myocardial lipid homeostasis. In this regard, upregulated sarcolemmal FAT/CD36 expression is associated with enhanced cardiac FA uptake, lipid accumulation, and consequent cardiac dysfunction [55,56], and inhibition of FAT/CD36 suppresses FA uptake [57], suggesting that upregulation of cardiac FAT/CD36 is a key factor that contributes to lipotoxicity. Therefore, current data suggest that IL-6 deficiency may augment myocardial lipid uptake by upregulating FAT/CD36 expression, which thereby aggravates cardiac lipotoxicity.

**Figure 2:**
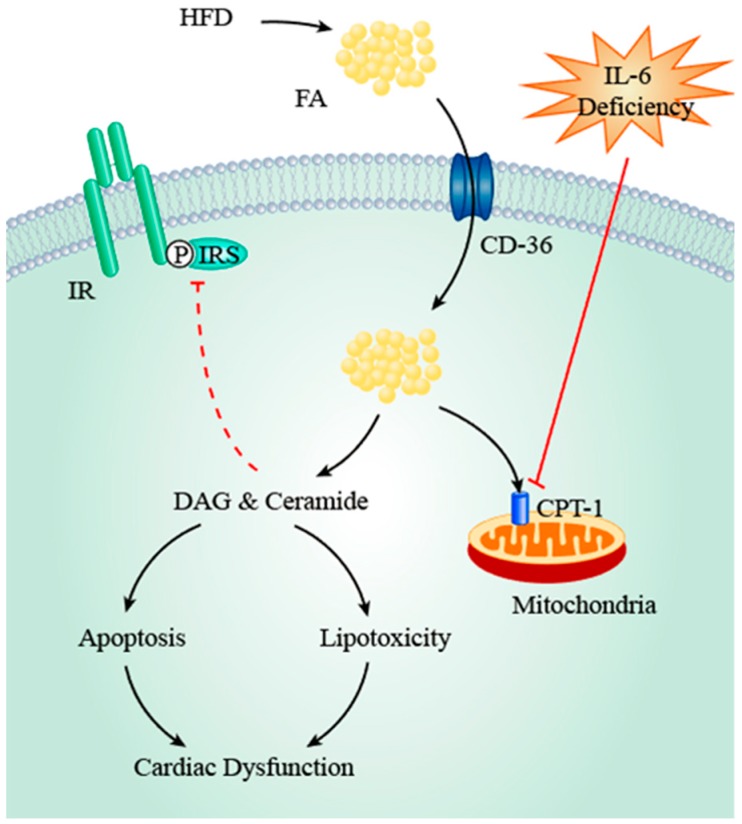
The role of IL-6 deficiency in intramyocardial fatty acid accumulation. IL-6 deficiency upregulates CD36 which is the key protein carrier of fatty acid across plasma membrane, thereby enhancing myocardial fatty acid uptake and accumulation. Moreover, IL-6 deficiency inhibits CPT1 which is involved in fatty acid translocation into mitochondria, thereby exacerbating fatty acid excess. Due to the limited storage capacity, fatty acid deposits are converted into toxic lipid metabolites (e.g., DAG and ceramide) which result in cardiac insulin resistance and cellular apoptosis. Taken together, fatty acid per se and its metabolites lead to myocardial lipotoxicity. Black arrows: direct stimulatory effects or consequences of upstream factors; Red dashed arrows: multistep inhibitory effects; Red solid arrows: direct inhibition. FA, fatty acid; HFD, high-fat diet; DAG, diacylglycerol; CPT-1, Carnitine palmitoyltransferase I; IR, insulin receptor; IRS-1, insulin receptor substrate 1.

Cardiac lipid overload impairs myocardial function and predisposes to lipotoxicity by several mechanisms. FA is the endogenous ligand of Peroxisome Proliferator-activated Receptor (PPAR) and its activation results in upregulation of enzymes involved in FA β-oxidation, with reciprocal downregulation of enzymes for glucose metabolism [58]. The consequent stimulation of FA metabolism and repression of glucose utilization may lead to ventricular hypertrophy and systolic dysfunction [58]. Furthermore, myocardial lipid deposits are converted into toxic metabolites (e.g., DAG and ceramide) due to limited storage capacity. DAG activates protein kinase C θ (PKC θ) and results in cardiac insulin resistance [59], which in turn potentiates cardiac lipotoxicity by repression of glucose metabolism and concomitant stimulation of FA oxidation. Furthermore, PKC activation indirectly stimulates the NF-κB (Nuclear Factor-κB) pathway which upregulates expression of enzymes involved in ceramide synthesis (e.g., STP (serine palmitoyltransferase) and CerS (Ceramide synthase)) [60,61,62]. The resultant elevation of ceramide levels in turn leads to apoptosis of cardiomyocytes and myocardial insulin resistance [23]. Taken together, recent studies indicate that accumulation of both FA *per se* and its metabolites as a result of IL-6 deficiency negatively impacts cardiac function and leads to lipotoxicity (Figure 2).

### 4.2. IL-6, PPAR & PGC-1α and Mitochondria 

PPARs are nuclear receptors which are activated by various ligands including FA. Once activated, PPARs undergo heterodimerization with retinoid X receptor and bind to PPAR response elements present within the promoter region of target genes, thereby regulating their expression. PPARα and PPARβ/δ are highly expressed in cardiac muscle whereas PPARγ is present in abundance in adipose tissue [63,64]. PPARα regulates expression of protein transporters and enzymes involved in FA uptake and oxidation. PPARγ coactivator 1α (PGC-1α) is the coactivator of PPAR and has implications in mitochondria biogenesis by stimulating various transcription factors including nuclear respiratory factors-1 (NRF-1) and nuclear respiratory factors-2 (NRF-2) which in turn upregulate MTFA (also known as Tfam or TCF6, mitochondrial transcription factor A). MTFA promotes replication and transcription of mitochondria DNA (mtDNA) by interacting with proteins encoded by target genes of NRF-1 and NRF-2, thereby facilitating mitochondrial biogenesis [65]. Endonuclease G (EndoG) is another protein implicated in mitochondrial biogenesis by facilitating maturation of RNA primers for DNA polymerase γ, thereby initiating mtDNA replication [66]. 

Several lines of evidence suggest the regulatory role of IL-6 in cardiac PPAR and PGC-1α expression. PPARα protein levels in cardiomyocytes of WT mice are significantly higher than those of IL-6^−/−^ mice and a trend of decreased PPARα mRNA level in IL-6^−/−^ mice was observed, thereby suggesting a positive link between IL-6 and PPARα [67]. Our group also demonstrated that IL-6 deficiency results in decreased mRNA levels of PPARα in mice during HFD feeding [36]. Deficiency of PPARα leads to downregulation of enzymes involved in FA β-oxidation and reduced rate of FA oxidation [68], which in turn exacerbates intracellular accumulation of FA and lipotoxicity [69]. Furthermore, Bonda et al. showed that lower cardiac content of lipid droplets in IL-6^−/−^ mice is possibly due to FA esterification and storage as a result of PPARα deficiency [67]. Since intracellular FA is deleterious to cardiac function due to its pro-apoptotic and protonophoric actions, lipid droplets may protect cardiomyocytes against such toxic effects by sequestrating cytosolic FA and functioning as an inert pool [70]. Therefore, it is postulated that downregulation of PPARα induced by IL-6 deficiency results in a reduced lipid droplets amount, thereby providing another mechanism of cardiac lipotoxicity induced by IL-6 deficiency [67]. That said, cardiac FA storage capacity is limited and FA accumulation beyond this threshold is thus toxic.

Moreover, cardiac PGC-1α levels decrease in both WT and IL-6^−/−^ mice fed HFD. However, the magnitude of reduction is less pronounced in WT mice, thereby suggesting that IL-6 partially protects against suppressed expression of PGC-1α induced by obesity [67]. Decreased expression of PGC-1α in Drosophila results in reduced inhibition of FA synthase, thereby leading to cardiac TAG accumulation and lipotoxicity which are reversed by overexpression of PGC-1α [71]. Moreover, activation of myocardial PGC-1α by overexpression of pyruvate dehydrogenase kinase 4 stimulates FA β-oxidation and reduces lipid deposits during HFD feeding [72]. Furthermore, our group reported a slight but significant increase in expression of genes related with mitochondria biogenesis (i.e., *Pgc-1α*, *Endog*, and *Mtfa*) in WT mice compared with IL-6^−/−^ mice when feeding HFD, suggesting that IL-6 deficiency impairs mitochondria synthesis [36]. As mitochondria are the sites where FA β-oxidation occurs, enhanced mitochondria biogenesis potentiates FA oxidation capacity and protects against lipid accumulation and lipotoxicity [73]. Recent studies in skeletal muscle revealed that stimulation of FA β-oxidation alleviates intracellular lipid accumulation and enhances insulin sensitivity [74,75], thereby suggesting similar protective roles of IL-6 in cardiac muscle. However, inconsistency remains as other researchers demonstrated that enhanced FA β-oxidation results in elevated production of acetyl-CoA which inhibits pyruvate dehydrogenase and eventually leads to cardiac insulin resistance [76,77].

**Figure 3:**
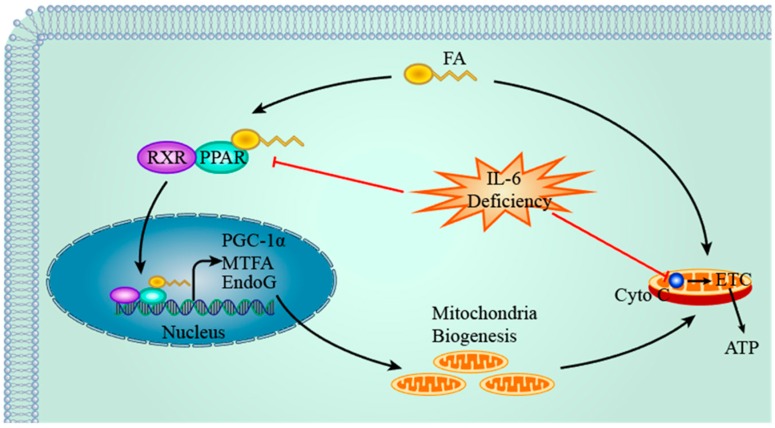
The role of IL-6 deficiency in mitochondria dysfunction. IL-6 deficiency negatively regulates PPARα, which results in downregulation of proteins involved in mitochondria biogenesis (e.g., PGC-1α, MTFA, and EndoG) and enzymes of fatty acid oxidation. Furthermore, IL-6 deficiency inhibits expression of cytochrome c, which is involved in the electron transport chain, thereby resulting in cardiac ATP deficiency. Taken together, IL-6 deficiency impairs mitochondria biogenesis as well as fatty acid oxidation, which leads to cardiac energy deficit. Red arrows indicate inhibitory effects of IL-6 deficiency and black arrows represent stimulatory effects or consequences of upstream factors. FA, fatty acid; RXR, retinoid X receptor; PPAR, Peroxisome Proliferator-activated Receptor; PGC-1α, PPARγ coactivator 1α; MTFA, mitochondrial transcription factor A; EndoG, Endonuclease G; Cyto C, cytochrome C; ETC, electron transport chain.

In addition, IL-6 deficiency negatively impacts mitochondrial oxidative phosphorylation (OXPHOS). Mitochondrial cytochrome c (cyto c) is an indicator of OXPHOS efficiency as it is involved in electron transport chain. HFD induces reduced expression of cardiac cyto c in both WT and IL-6^−/−^ mice; however, the decrease in IL-6^−/−^ mice is more prominent than that in WT mice, suggesting that IL-6 plays a protective role against mitochondria dysfunction [67]. One possible explanation is that IL-6 deficiency reduces PPAR and PGC-1α levels and this in turn inhibits mitochondrial biogenesis, which results in decreased mitochondria amount as reflected by lower cyto c levels and, hence, OXPHOS. However, no significant differences of mitochondrial protein cytochrome c oxidase and citrate synthase levels, which also reflect mitochondria amount, were observed between WT and IL-6^−/−^ mice when feeding HFD [67], thereby suggesting that the preservative effects of IL-6 on cyto c are not secondary to the impacts on mitochondrial biogenesis. Consequently, decreased OXPHOS leads to accumulation of acylcarnitine when FA β-oxidation rate remains unaltered [78]. Acylcarnitine, a cardiac lipotoxin, consequently perturbs sarcolemmal integrity and electrophysiological properties [79], thereby predisposing to cardiac dysfunction and lipotoxicity (Figure 3).

However, discrepancy exists with regards to the positive correlation between IL-6 and PPAR. Haffar and colleagues observed that palmitate treatment of rat neonatal cardiomyocytes induces an early increase in PPAR activity measured by mRNA levels of PPAR target genes, which is followed by a later decrease [80]. The reduction phase is supported by decreased protein levels of PPAR and suppressed expression of its target gene *Cpt1*. Furthermore, palmitate-induced IL-6 expression precedes the reduction of PPAR activity. Therefore, these authors proposed that palmitate acts as PPAR ligand and thus is responsible for the early increase in activity, whereas IL-6 potentially contributes to degradation of PPAR and the later decreased activity [80]. However, another possible explanation for the later decrease is the existence of a negative feedback loop. In this regard, target gene expression facilitates PPAR degradation, as evidenced by decreased PPAR protein levels during the later phase. Although other studies involving adipocytes and hepatocytes demonstrated that IL-6 treatment leads to reduced expression and activity of PPAR [81,82], it remains unclear until further validation is given by studies using cardiomyocytes treated with IL-6. Taken together, current evidence suggests that IL-6 preserves FA oxidation and mitochondrial biogenesis by maintaining cardiac PPAR and PGC-1α expression, which is beneficial to cardiac function.

### 4.3. IL-6, AMPK and ACC

AMP-activated protein kinase (AMPK) is a key cellular energy sensor which is activated by increased concentration of AMP. AMPK activation elicits various downstream events, with the net effect being an enhanced production of ATP to meet energy demand. In myocardium, a high AMP/ATP ratio leads to phosphorylation of AMPK which in turn stimulates glucose uptake and glycolysis, as well as FA oxidation [83]. One of the key targets regulated by AMPK is acetyl-CoA carboxylase (ACC) which is responsible for catalyzing the conversion of acetyl-CoA to malonyl-CoA. Therefore, AMPK also regulates FA *de novo* synthesis by phosphorylating ACC and thus controls the rate-limiting step (i.e., conversion of acetyl-CoA to malonyl-CoA) in a series of reactions leading to FA production.

**Figure 4:**
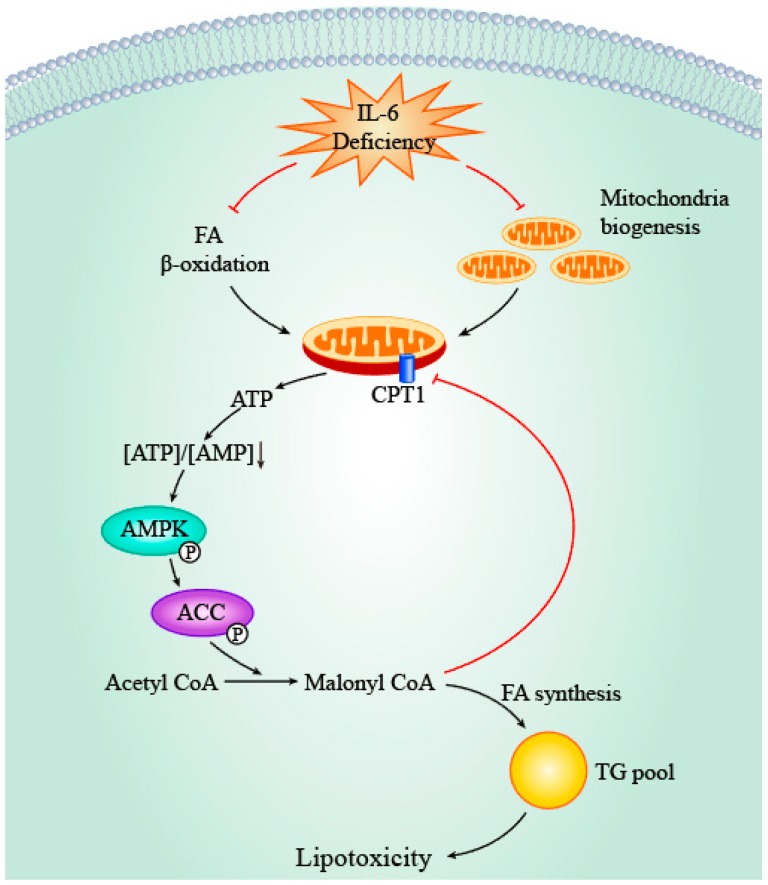
The role of IL-6 deficiency in the AMPK/ACC axis. IL-6 deficiency inhibits FA oxidation and mitochondria biogenesis through multistep pathways, thereby resulting in cardiac ATP depletion. Consequently, reduced [AMP]/[ATP] ratio activates AMPK and subsequently stimulates ACC which in turn catalyzes the conversion of acetyl CoA to malonyl CoA. As this reaction is the rate-limiting step in FA *de novo* synthesis, activation of ACC promotes TG pool expansion and lipotoxicity. Moreover, malonyl CoA inhibits CPT1 which is responsible for translocation of activated FA into mitochondria for oxidation, thereby exacerbating lipid accumulation and lipotoxicity. Black arrows denote stimulatory or consequential effects of upstream factors and red arrows indicate inhibitory effects. FA, fatty acid; CPT1, carnitine palmitoyltransferase-1 (CPT-1); AMPK, AMP-activated protein kinase; ACC, acetyl-CoA carboxylase; TG, triglycerides.

Several studies suggest the potential regulatory role of IL-6 in the AMPK–ACC axis. The majority of these studies utilized non-cardiac tissue (e.g., skeletal muscle and adipose tissue) and indicate that IL-6 treatment stimulates AMPK and ACC phosphorylation [84]. Exercise stimulates IL-6 synthesis in contracting muscle and, therefore, the same effects were reproduced in gastrocnemius muscle from exercise-trained mice [85]. However, no significant difference of AMPK phosphorylation levels was observed in left ventricle cardiac tissue between WT and IL-6^−/−^ mice after treadmill training [85]. That said, the possibility that the AMPK–ACC axis is regulated by cellular responses secondary to IL-6 cannot be excluded. In this regard, our group showed markedly enhanced phosphorylation of cardiac AMPK and ACC in IL-6^−/−^ mice in comparison with WT mice during HFD feeding [36]. As discussed above, we noticed that IL-6 deficiency negatively regulates FA oxidation and mitochondrial biogenesis, which in turn leads to depletion of ATP and accumulation of AMP. Thus, we propose that the enhanced AMPK phosphorylation results from the indirect effects of IL-6 deficiency. Taken together, current evidence suggests that IL-6 may regulate the AMPK–ACC axis in an indirect manner via multiple downstream factors which converge on the AMP/ATP ratio, although additional research is required (Figure 4).

The proposed indirect activation of AMPK by IL-6 deficiency is suggested to play a protective role for the heart against lipotoxicity. Inhibition of FA synthesis by ACC phosphorylation may reduce synthesis of TG, and, in turn, the risk of cardiac lipid accumulation and consequent lipotoxicity. Moreover, reduced production of malonyl-CoA due to ACC phosphorylation removes the inhibitory effects on carnitine palmitoyltransferase-1 (CPT-1), which in turn enhances mitochondrial FA uptake and oxidation [86,87], which protects cardiomyocytes against lipotoxicity by facilitating clearance of intracellular lipid. One theoretical concern is that elevated AMPK phosphorylation was reported to induce cardiac apoptosis [88] and autophagy [89]. However, these effects were not observed in our study [36], thereby supporting the hypothesis that AMPK phosphorylation as a result of secondary effects of IL-6 deficiency might play a beneficial role in protecting against cardiac dysfunction. Of note, this may help explain the apparent discrepancy between the protective effects of IL-6 treatment in mitochondrial biogenesis and function and the beneficial roles of IL-6 deficiency in TG pool expansion. In this regard, IL-6 deficiency impairs cardiac FA consumption, while this in turn activates the intrinsic protective mechanism by suppressing de novo FA synthesis.

## 5. Conclusions

The pleiotropic cytokine IL-6 is involved in lipid metabolism in both humans and rodents despite its key role during inflammation. Since the high energy demand of a well-perfused heart is met primarily by FA β-oxidation, IL-6 is implicated in cardiac lipotoxicity. The function of IL-6 in the process of cardiomyocyte metabolic homeostasis is gradually becoming more clearly understood. Current studies suggest that IL-6 deficiency results in cardiac lipotoxicity by deleterious effects on intracellular lipid accumulation and, thus, generation of toxic lipid metabolites, thereby precipitating cardiac dysfunction. However, many details in these processes remain unknown. Are there other molecules that regulate IL-6 and its targets during HFD-induced cardiac lipotoxicity? Are there more downstream molecules regulated by IL-6 in the process of cardiac FA oxidation? The molecular mechanism of IL-6 in cardiac FA metabolism is not fully understood yet, but it is important to address it due to the important roles in development of various cardiomyopathy. Decoding the activators and effectors of IL-6 in cardiac lipotoxicity during HFD-induced obesity will provide cues for treatment of obesity-associated dyslipidemia and cardiac lipotoxicity, and improve development of novel drug therapies.

## Figures and Tables

**Table 1:** The role of interleukin-6 (IL-6) in metabolic regulation in humans.

Subject/Description	Treatment	Observed Effects	Reference
Human/healthy males	rhIL-6 infusion for 4 h	Increase of lipolysis in skeletal muscle; Increase of systemic FA oxidation	[20]
Human/healthy males	IL-6 infusion for 2.5 h	Net increase of glycerol from subcutaneous adipose tissue; Increased uptake of FA and glycerol in splanchnic regions	[24]
Human/healthy males	IL-6 infusion for 4 h	Increased FA oxidation	[25]
Human/males with T2D vs. control	rhIL-6 infusion for 3 h	Increase of palmitate *R*_a_ and *R*_d_ in both groups	[26]
Human/healthy males	IL-6 infusion for 3 h	Increase of serum FA levels; Increased *R*_a_ of endogenous FA; Enhanced systemic FA oxidation	[27]
Human/patients with multicentric Castleman disease	Treatment of humanized anti-human IL-6 receptor monoclonal antibody	Gain of body weight; Hypertriglyceridemia; Hypercholesterinemia	[28]
Human/healthy females with G or C alleles at position 174 of *IL-6* gene	n.d.	Trend of increased plasma IL-6 levels and elevated serum TG, VLDL-C and FFA in IL-6 G174C polymorphism	[29]

rhIL-6, Recombinant human IL-6; FA, Fatty acid; *R*_a_, Rate of appearance; *R*_d_, Rate of disappearance; n.d., No data; TG, Triglyceride; VLDL-C, Very-low-density lipoprotein cholesterol; FFA, Free fatty acid.

**Table 2:** The role of IL-6 in metabolic control in rodents.

Species/Description	Treatment	Observed Effects	Reference
Mice/IL-6 KO	n.d.	Mature-onset obesity: Increased weight of subcutaneous fat pad	[18]
Mice/IL-6 KO; HFD	Intracerebroventricular IL-6 injection for 2 weeks	Decreased relative weight of mesenteric and retroperitoneal fat pads; Suppressed body weight	[32]
Mice/HFD; IL-6 transgenic mice (with sustained release of human IL-6)	n.d.	Decreased food intake; Increased energy expenditure; Reduced visceral fat on normal chow and free from HFD-induced obesity	[33]
Rat/male	Direct delivery of recombinant adeno-associated viral vector expressing murine IL-6 into hypothalamus	Suppressed weight gain and visceral adiposity	[34]
Mice/HFD-induced obese mice	Delivery of pLIVE-IL-6 plasmid expressing murine IL-6	Reduction in body weight; Increased expression of enzymes involved in FA oxidation	[19]
Mice/Double transgenic mice co-expressing IL-6 and soluble IL-6 receptors	n.d.	Reduced body weight; Decreased body fat	[35]
Mice/IL-6 KO; female; HFD	n.d.	Decreased body weight gain and fat mass	[36]

KO, Knock out; HFD, High-fat diet; n.d., No data.

## Abbreviations

IL-6	Interleukin-6
FA	Fatty acid
rhIL-6	Recombinant human IL-6
VLDL	Very low-density lipoprotein
HFD	High-fat diet
sIL-6R	Soluble IL-6 receptor
TAG	Triacylglycerol
LPL	Lipoprotein lipase
mtDNA	Mitochondria DNA
TG	Triglycerides
PPAR	Peroxisome Proliferator-activated Receptor
PGC-1α	PPARγ coactivator 1α
FAT/CD36	Fatty acid translocase
WT	Wild-type
FABPpm	Plasma membrane isoform of fatty acid binding protein
FATP	Fatty acid transport protein
PKC θ	Protein kinase C θ
NRF	Nuclear respiratory factor
AMPK	AMP-activated protein kinase
CPT-1	Carnitine palmitoyltransferase-1
